# Advances in Aptamer-Based Biosensors and Cell-Internalizing SELEX Technology for Diagnostic and Therapeutic Application

**DOI:** 10.3390/bios12110922

**Published:** 2022-10-25

**Authors:** Zixuen Gan, Muhamad Aidilfitri Mohamad Roslan, Mohd Yunus Abd Shukor, Murni Halim, Nur Adeela Yasid, Jaafar Abdullah, Ina Salwany Md Yasin, Helmi Wasoh

**Affiliations:** 1 Faculty of Biotechnology and Biomolecular Sciences, Universiti Putra Malaysia, Serdang 43400, SGR, Malaysia; 2 Faculty of Science, Universiti Putra Malaysia, Serdang 43400, SGR, Malaysia; 3 Aquatic Animal Health and Therapeutics Laboratory, Institute of Bioscience, Universiti Putra Malaysia, Serdang 43400, SGR, Malaysia; 4 Halal Products Research Institute, Universiti Putra Malaysia, Serdang 43400, SGR, Malaysia

**Keywords:** aptamer, SELEX, pathogenic microorganisms, mammalian cells, cell internalization, biosensors

## Abstract

Aptamers are a group of synthetic single-stranded nucleic acids. They are generated from a random library of single-stranded DNA or RNA by a technology named systematic evolution of ligands by exponential enrichment (SELEX). SELEX is a repetitive process to select and identify suitable aptamers that show high affinity and specificity towards target cells. Great strides have been achieved in the design, construction, and use of aptamers up to this point. However, only a small number of aptamer-based applications have achieved widespread commercial and clinical acceptance. Additionally, finding more effective ways to acquire aptamers with high affinity remains a challenge. Therefore, it is crucial to thoroughly examine the existing dearth and advancement in aptamer-related technologies. This review focuses on aptamers that are generated by SELEX to detect pathogenic microorganisms and mammalian cells, as well as in cell-internalizing SELEX for diagnostic and therapeutic purposes. The development of novel aptamer-based biosensors using optical and electrical methods for microbial detection is reported. The applications and limitations of aptamers are also discussed.

## 1. Introduction

Aptamers are a group of short single-stranded nucleic acids (ssDNA or ssRNA), usually shorter than 40 nucleotides [1]. In the process of aptamer selection, DNA is more stable if compared to RNA as RNA aptamers are more susceptible to cellular nucleases [2]. Aptamers are widely applied in medical science and molecular biology fields as they have high affinity and specificity to target molecules for diagnostic or therapeutic purposes. Aptamers are emerging tools of biomarkers for clinical diagnostics and therapeutic agents for targeted therapy [3]. Examples of aptamer-binding targets are bacteria, amino acids, and small metal ions [4]. Recent research has employed aptamers as an alternative to antibodies in the detection of histone-modifying enzyme activity, providing a new platform for clinical diagnosis [5]. Compared to antibodies, aptamers are easier and more reliable for high-quality production through chemical synthesis, because they are more stable against harsh conditions such as heat denaturation [1]. Aptamers also have high affinity with dissociation constants (Kd) in the range from nanomolar to picomolar [6]. Besides that, they have added features such as low molecular weight, easy modification, nontoxicity, and low immunogenicity that make them a preferred choice compared to antibodies [7].

**Figure 1:**
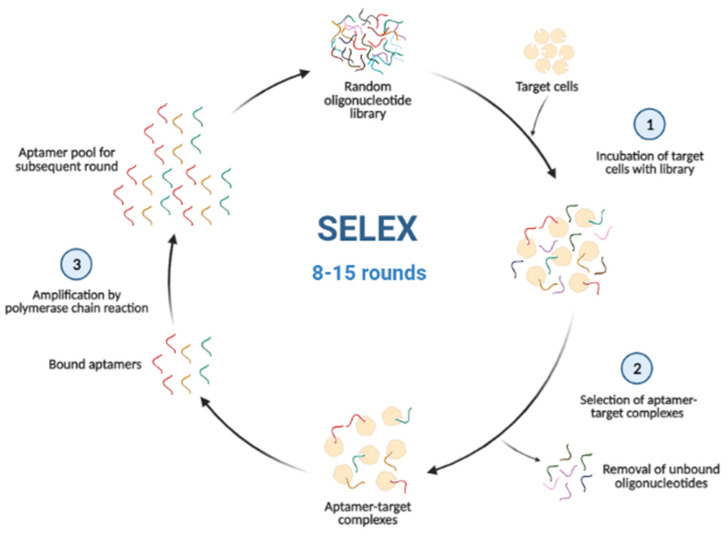
A general scheme of SELEX protocol. (Created in BioRender.com, accessed on 9 September 2022).

Aptamers are identified by performing an iterative selection–amplification process known as systematic evolution of ligands by exponential enrichment (SELEX), which was first developed by Ellington and Szostak and Tuerk and Gold separately in 1990 [8,9,10]. A general SELEX methodology (Figure 1) consists of three major stages: incubation of target cells with the library, selection of aptamer–target complexes from unbound oligonucleotides, and the amplification of bound sequences [11]. In this process, a random library is required, composed of single-stranded nucleic acids (DNA or RNA), which are flanked at both ends with specific sequences as primer binding sites for polymerase chain reaction (PCR) [12]. During the selection process, the target of interest is incubated into the random pool, and then variants of nucleic acid that bind to the target molecule are chosen, while unbound variants are eliminated [13]. The target-bound sequences undergo an amplification process by PCR or reverse-transcription PCR for DNA or RNA sequences, respectively [10]. The amplified aptamer pool will be used for the subsequent round of SELEX. To identify the most suitable aptamer, the SELEX process is usually repeated for 8–15 rounds [13]. The obtained aptamer pool during the final round will be analyzed to determine the sequence and structure of the selected aptamers after the SELEX protocol. In this review, research studies related to aptamers selected against pathogenic microorganisms and mammalian cells and their applications in biosensor platforms are highlighted. Importantly, we also briefly discuss the SELEX process and cell-internalizing SELEX-generated aptamers.

## 2. Aptamers against Pathogenic Microorganisms and Mammalian Cells

The SELEX process can be conducted to target pathogenic microorganisms such as bacteria, viruses, and protozoa for the diagnosis of infectious diseases caused by pathogens [14]. Aptamers generated through this process are also targeting mammalian cell lines [15]. Aptamers identified from SELEX can target specific surface molecules on pathogens or cell lines with high affinity and inhibit the mechanism of disease-causing agents. These antibacterial or antiviral aptamers have potential applications to provide a solution for rapid diagnosis and early treatment of diseases.

### 2.1. Aptamers against Pathogenic Bacteria

Various aptamers have been reported to have great potential for the detection of pathogenic bacteria. *Escherichia coli* O157:H7 strain is a foodborne pathogen that can cause such diseases as diarrhea and kidney failure [11]. Research by Amraee et al. identified a specific DNA aptamer from a single-stranded DNA (ssDNA) library to detect the *E. coli* O157:H7 strain [7]. A dissociation constant (Kd) value of 107.6 ± 67.8 pM was calculated from the selected aptamer. Another work by Yu et al. also employed the *E. coli* O157:H7 strain as the target bacteria [6]. The selected ssDNA aptamer was further enhanced with a quartz crystal microbalance (QCM) sensor for more rapid and sensitive detection of the pathogen at low concentrations. The aptamer gave a dissociation constant of 10.30 nM in this study.

*Salmonella typhimurium* is another example of a foodborne pathogen that causes salmonellosis that affects the intestinal tract in humans and animals [16]. An ssDNA aptamer named ST2P binding to the bacteria showed a high affinity with a Kd value of 6.33 ± 0.58 nM [17]. The ST2P aptamer was applied in a fluorescent bioassay to enhance the detection of *S. typhimurium*. Besides selecting aptamers against a single target, Park et al. discovered ssDNA aptamers (Se1, Se2 and St1) targeting two bacteria, which were *S. typhimurium* and *Salmonella enteritidis* [18]. The three aptamers had dissociation constants in the nanomolar (nM) to micromolar (µM) range, where they had potential use for the sensitive detection of salmonellosis.

There are also aptamers developed against *Mycobacterium tuberculosis*, an airborne pathogen causing tuberculosis, a pulmonary disease [19]. From a study by Chen et al., an ssDNA aptamer (NK2) targeting the *M. tuberculosis* H37Rv strain was identified [20,21]. The NK2 aptamer was found to have inhibitory effects on the bacteria in the mouse model, where the function of the aptamer was further investigated to develop it as a therapeutic agent against tuberculosis.

### 2.2. Aptamers against Pathogenic Viruses

Some aptamers are applied in the development of detection assays for pathogenic viruses. Aptamers were studied to target the H9N2 avian influenza virus (AIV) that threatens the poultry industry and public health [22]. Two DNA aptamers (A9 and B4) were selected through the capillary electrophoresis-based SELEX procedure. The aptamers targeting hemagglutinin (HA) surface protein and also the whole virus had dissociation constants in the nanomolar (nM) range, and they were found to have inhibitory effects on the H9N2 virus-infected cells. In a previous study by Kwon et al., an RNA aptamer (HA12-16) targeting glycosylated HA protein of the H5N1 avian influenza virus was isolated [23]. It was also found that the specific aptamer was able to suppress viral infection in host cells, thus having the potential to be developed as an antiviral agent for therapeutic purposes.

Apart from the influenza virus, aptamers have been developed to target the human immunodeficiency virus (HIV). The virus is the causative agent of acquired immunodeficiency syndrome (AIDS), which is fatal, thus the development of antiviral drugs is crucial [11]. Several aptamers have been reported to have inhibitory effects on the virus by targeting binding sites such as Tat protein [24], glycoprotein gp120 [25] and CD4 receptor [26]. For example, in the study by Mufhandu et al., the researchers isolated an RNA aptamer (UCLA1) that can bind tightly to glycoprotein gp120 of HIV-1 subtype C [25]. The aptamer was observed to have an average dissociation constant of 0.15 nM and was able to neutralize the infectivity of HIV-1.

Recently, aptamers have been extensively studied for their detection of the severe acute respiratory syndrome coronavirus 2 (SARS-CoV-2). It is of the utmost importance to seek new molecular tools for the diagnosis and treatment of the fatal COVID-19 disease caused by the virus. Research by Valero et al. reported a serum-stable 2′-fluoro RNA aptamer (RBD-PB6) that binds to the receptor binding domain (RBD) of SARS-CoV-2 spike protein [27]. They further performed aptamer multimerization, which increases the binding affinity to a low picomolar range and the aptamer is capable of neutralizing SARS-CoV-2 infection in cells. Another study by Yang’s group identified six novel DNA aptamers against SARS-CoV-2 by blocking the RBD of spike protein subunit 1 [28]. The aptamers (nCoV-S1-Apts) had dissociation constants in the range of 0.118 ± 0.033 to 85.610 ± 14.219 nM. Aptamers that target the spike protein of the virus are important to inhibit the infection cycle, as the interaction between the spike protein and the angiotensin-converting enzyme 2 (ACE2) receptor of the cell surface is prohibited.

There are also pathogenic viruses that affect the aquaculture industry by causing serious fish diseases. Aptamers have been applied in rapid-detection assays for aquatic viruses [16]. Several studies have reported the application of DNA or RNA aptamers that target different aquatic viruses, such as the Hirame rhabdovirus (HIRRV) [29], Singapore grouper iridovirus (SGIV) [30] and red-spotted grouper nervous necrosis virus (RGNNV) [31]. However, if compared to the number of aptamers targeting human viruses, there are fewer aptamers that have been reported to detect aquatic viruses [16].

### 2.3. Aptamers against Pathogenic Unicellular Parasites

Unicellular parasites can cause such diseases as malaria and Chagas disease [11]. There are only several aptamers found to be against parasites, which include *Leishmania-*, *Plasmodium-* and *Trypanosoma*-specific aptamers [16]. Previously, Ulrich’s group obtained RNA aptamers using *Trypanosoma cruzi* as the target, a live pathogenic American trypanosome that causes Chagas disease, an inflammatory and infectious disease in humans [32]. Based on the research, the generated aptamers were obtained from a 2′-fPy-RNA pool, and they competed with the host cell–matrix molecules to bind to the pathogen with affinity of 40–400 nM.

Another study by Barfod’s group obtained 2′-fluoro RNA aptamers targeting the malaria plasmodium *Plasmodium falciparum* [33]. The aptamers were selected against the erythrocyte membrane protein 1 of *P. falciparum*, which is responsible for the pathogenicity of the parasite. The aptamers could be developed into antimalaria drugs, and their ability to recognize the parasite could help in malaria diagnostics. Table 1 below summarizes several examples of pathogenic microorganisms that have been targeted by DNA or RNA aptamers.

### 2.4. Aptamers against Mammalian Cells

Apart from pathogenic microorganisms, SELEX is also performed to generate aptamers against mammalian cells. Aptamers have been used as therapeutic agents in cancer treatment by binding to target molecules to inhibit their activities [45,46]. For instance, aptamers were generated to target antigens expressed by a monolayer of tumor cells [47]. The research was conducted by using a glioblastoma cell line, U251, as the target for a SELEX library of single-stranded DNA. In some cases, there are two types of cells, which are the specific cancer cell types, and the normal cells or other cancer cell types that are used in the SELEX process as targets and controls, respectively. A work conducted by Shangguan’s group utilised human precursor T-cell acute lymphoblastic leukemia cells (CCRF-CEM) and the human B-cell line from Burkitt’s lymphoma (Ramos) for positive and negative selection, respectively, in the SELEX process to generate aptamers as cell-specific molecular probes [48].

In addition, aptamer-based molecular tools are major contributions to the discovery of cancer biomarkers by high-affinity recognition of membrane protein receptors, growth factors and transcription factors [49]. Research by Mahlknecht’s group generated a 14-nucleotide aptamer against a biomarker for native human epidermal growth factor receptor 2 (HER2) protein [50]. The conjugation of the aptamer with HER2 protein, which is overexpressed in tumor cells, including breast and gastric tumors, managed to suppress the growth of tumor cells. Most of the aptamers targeting HER2 protein were used to deliver chemotherapeutics or nanoparticles to cancer cells for the treatment of HER2-overexpressing cancers [46].

Virus-infected cells, for example, vaccinia virus-infected adenocarcinoma epithelial cells (A549) were used by Tang et al. in SELEX for the generation of DNA aptamers that can act as molecular probes towards virus-infected cells [51]. Besides that, somatic cells and embryonic stem cells (ESCs) can be used as target molecules, such as using mouse ESCs to generate 2′-fPy-RNA aptamers [52].

Furthermore, aptamers can be labeled with fluorescent particles to serve as cancer probes targeting cancer cell lines [53]. In a study by Wang et al., a novel aptamer for the recognition of U-2 OS human osteosarcoma cell line was designed using the SELEX technique [54]. The aptamer was identified from nine rounds of selection in the SELEX process followed by sequence analysis and fluorescent labeling. The selected aptamer showed a high binding affinity towards U-2 OS target cells instead of non-osteosarcoma negative-control tumor cell lines.

## 3. Cell-Internalization SELEX

**Figure 2:**
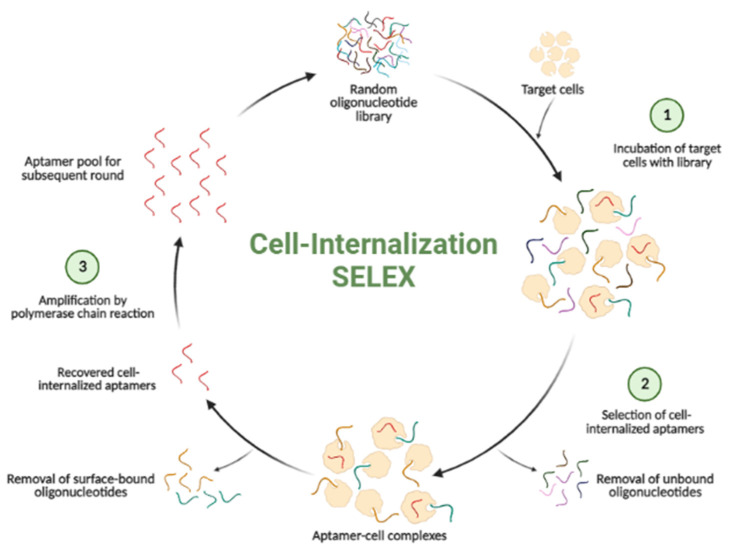
A schematic illustration of cell-internalization SELEX. (Created in BioRender.com, accessed on 9 September 2022).

Apart from aptamers that bind to molecules expressed on the surface of target cells, some aptamers can be intracellularly transported into living cells through endocytosis [55]. The difference in this SELEX process (as shown in Figure 2) if compared to cell-based SELEX is that not only unbound sequences are discarded but also surface-bound sequences to target cells [15]. These intracellular targeting aptamers are vital in drug delivery and targeted therapeutic application towards diseases such as Alzheimer’s disease and cancers [55].

### 3.1. Cell-Internalizing DNA Aptamers

Research by Wu et al. aimed to identify aptamers that specify intracellular transport [56]. The variants that were taken up by the target cells were then recovered by cell lysis with proteinase and detergent. From this work, they managed to identify DNA motifs that can be transported into human chronic lymphocytic leukemia B cells. Another study by Ranches et al. identified cell-internalizing DNA aptamers targeting cytokine-stimulated cells for chronic kidney disease treatment [57]. The aptamers that were isolated from the endosomal fractions of these cells have shown high binding affinity and internalizing properties. They could serve as potential molecular probes for the disease to overcome the limitations of current diagnostic biomarkers, such as serum creatinine and urine albumin.

In recent research reported by Tanaka et al., cell-internalizing aptamers were successfully generated from an artificial DNA library [58]. Their research suggested that the cell-internalizing ability of aptamers could be improved by incorporating hydrophobic groups, such as aromatic rings, into nucleobases. A hydrophobic uracil derivative was introduced into the library, thus improving the cell-penetrating function of aptamers. The generated aptamers with hydrophobic base modification managed to deliver antisense oligonucleotides (ASOs) into adenocarcinoma human alveolar basal epithelial cells (A549 cells). Another modification done by Alamudi’s group was introducing an additional highly hydrophobic unnatural base on top of the four natural bases (A, C, G, T) in the library [15]. The identified DNA aptamers displayed high binding affinity towards breast cancer cells. Moreover, the internalizing action of the aptamers within the bound cells shows a promising property for cancer cell detection and therapy.

### 3.2. Cell-Internalizing RNA Aptamers

SELEX is also used to generate RNA aptamers that can be internalized into the target cells to deliver secondary reagents such as small interfering RNAs (siRNAs), besides binding to a particular target protein on the cell surface. The process identified cell-internalizing RNA aptamers targeting two distinct receptor tyrosine kinases [59,60]. An important “preclearing” step was carried out with control cells that lack the target receptor so that the sequences that were internalized by these cells other than the target receptor were removed. Besides that, Thiel’s group performed optimization on the cellular uptake mechanism by incubating the oligonucleotide library at 37 °C and washing using 0.5 M NaCl salt to remove unbound and surface-bound sequences. As a result, rapidly internalized aptamers were recovered while removing noninternalized and slowly internalized aptamers [61] (pp. 187–199).

Another study by Thiel’s group reported the ability of cell-internalizing RNA aptamers to deliver therapeutic siRNAs to the human epidermal growth factor receptor 2 (HER2)-expressing breast cancer cells [62]. The aptamers that were internalized into HER2 cells silenced the expression of the antiapoptotic gene Bcl-2, which was targeted by the delivered siRNAs. The gene silencing is important to sensitize these cancer cells to chemotherapy. This aptamer–siRNA conjugate demonstrated the potential of RNA-based reagents to be developed for the treatment of breast cancer.

## 4. Aptamer-Based Biosensors

**Figure 3:**
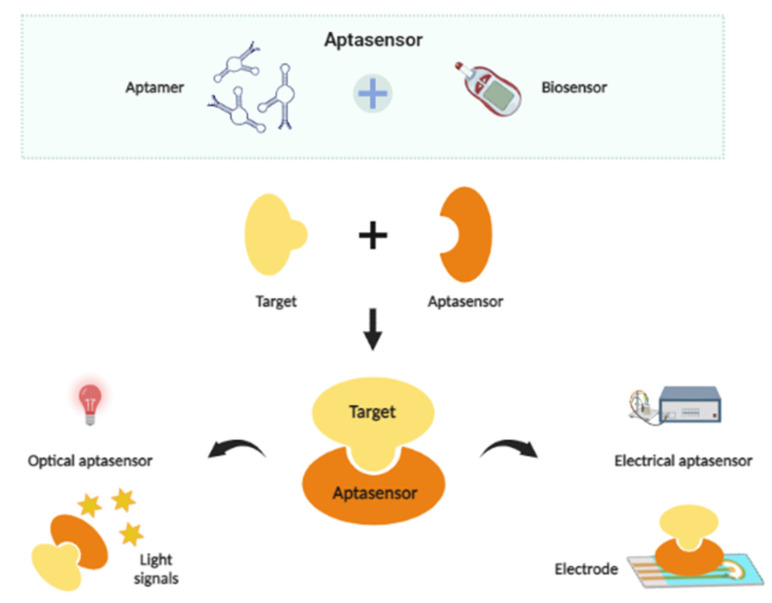
Simple principle of an aptasensor. (Created in BioRender.com, accessed on 9 September 2022).

The major components of a biosensor are a bioreceptor and a transducer. A few examples of bioreceptors commonly used in biosensor platforms are antibodies, cells, enzymes and nucleic acids [63]. In aptamer-based biosensors or aptasensors, aptamers are used as bioreceptors to identify and bind to the target organisms [64], while signal transducers translate and produce detectable signals resulting from the reaction between the target organism and bioreceptor [65]. Aptamer-based biosensors are categorized into optical and electrical aptasensors. The application of aptamers in biosensors has been widely applied in the detection of microorganisms such as bacteria or viruses. The simple principle of an aptamer-based biosensor is shown in Figure 3, and the types of detection methods by optical and electrical aptasensors are discussed to provide insight into their development strategies and detection capabilities in buffer medium.

### 4.1. Optical Aptasensors for Microbial Detection

Optical aptasensors apply optical principles for detection. The aptamers act as biorecognition elements with different optical principles, such as signal-transduction elements [66]. This group of aptasensors have advantages in cost-effectiveness, high sensitivity, rapid response and simple labeling [67]. A few approaches with optical aptasensors are fluorescence, chemiluminescence, surface plasmon resonance (SPR), surface-enhanced Raman scattering (SERS) and colorimetric [68].

Fluorescence is commonly used, as it is a simple approach with high sensitivity at a low cost. Fluorescent sensors produce an enhancement (signal-on) or a quenching (signal-off) effect that enables quantitative detection of the target [69]. According to Dwivedi et al. and Ohk et al., fluorescent particles are used by the fluorescent aptasensors as the output signals [70,71]. The signals are interpreted based on fluorescence intensity or fluorescence polarization. An example by Chung et al. showed that aptamer-conjugated fluorescent nanoparticles (A-FNPs) can detect *Escherichia coli* on an optofluidic particle-sensor platform [72]. By using the platform, they achieved a detection rate of about 100 *E. coli* single cells per second. Besides that, a fluorescent aptasensor was developed by Gao et al. to detect *Pseudomonas aeruginosa* in food samples (drinking water, orange juice and popsicle samples) [73]. Graphene oxide quantum dots were used to quench fluorescence signals produced by carboxyfluorescein-labeled aptamers. A linear relationship was shown between the fluorescence signals and *P. aeruginosa* concentration in the range of 1.28 × 10^3^–2.00 × 10^7^ cfu/mL with a detection limit of 100 cfu/mL.

Chemiluminescence is also an alternative to the fluorescence method, where the sensors emit light signals by energy from chemical reactions, providing cheap and fast detection [74]. Khang et al. applied guanine chemiluminescent reagents in the detection of *E. coli* O157:H7 with a 6-carboxyfluorescein (6-FAM)-conjugated aptamer [75]. The aptasensor was able to quantify the bacteria with a detection limit of 4.5 × 10^3^ cfu/mL, and the developed sensor was acceptable for the detection of *E. coli* in skim-milk samples. Apart from light signals, colorimetric analysis is a simple and low-cost technique based on direct observation of color change by the naked eye [76] or measurement using a spectrophotometer [13]. A colorimetric assay was used in aptasensors for microbial detection of *Salmonella typhimurium* [77], *Staphylococcus aureus* [78] and *Shigella flexneri* [79]. Gold nanoparticles (AuNPs) were used as colorimetric probes for the detection of *S. typhimurium* in milk samples by Duan’s group and *Sh. flexneri* in salmon samples by Feng’s group. In their research work, aptamers were immobilized onto the surface of AuNPs, whereby the complexes showed a linear response in the range of 25–10^5^ cfu/mL and 10^2^–10^6^ cfu/mL upon detection of *S. typhimurium* and *Sh. flexneri*, respectively, in buffer medium. On the other hand, Wang’s group applied copper-based metal–organic framework (Cu-MOF) nanoparticles as labels for colorimetric detection of *S. aureus* with a detection range of 50–10^4^ cfu/mL, and the detection was further applied in milk samples.

Besides that, SPR has been introduced as a label-free technique to determine affinity and kinetic parameters in biomolecular interactions. This technique works by measuring the changes in refractive index on a metal surface layer (gold, silver, or aluminum films) in response to the interaction between the immobilized aptamer on the surface and the target [80]. An application of SPR aptasensor is for the detection of avian influenza virus (AIV) H5N1 [81]. The biotinylated aptamers were immobilized on a sensor gold surface coated with streptavidin. The aptasensor could detect the virus with a detection range of 0.128–1.28 hemagglutination units (HAUs), then its applicability was tested using poultry swab samples. As for the SERS technique, it is based on the measurement of Raman signals produced during the interaction of target molecules and immobilized aptamers on the surface [82]. To enhance detection performance, the SERS method has also been combined with other techniques, such as colorimetric and fluorescence [68]. Based on Ma et al., a SERS-based aptasensor was invented to detect *S. typhimurium* in pork samples [83]. Spiny gold nanoparticles (SGNPs) were employed as the interface material, giving a linear range of 10^1^–10^5^ cfu/mL in a buffer medium. The limit of detection for this aptasensor was 4 cfu/mL. In earlier research by Zhang et al., a SERS- and nanoparticle-based aptasensor was constructed for simultaneous detection of *S. typhimurium* and *S. aureus* [84]. In the platform, the aptamers were conjugated with Fe_3_O_4_ magnetic gold nanoparticles (MGNPs) as the capture probe, while AuNPs were combined with Raman signal molecules and aptamers as the signal probe. The biosensor gave a linear detection range of 10^2^–10^7^ cfu/mL, with a detection limit of 35 cfu/mL and 15 cfu/mL for *S. aureus* and *S. typhimurium*, respectively, in a buffer medium.

### 4.2. Electrical Aptasensors for Microbial Detection

Electrical aptasensors produce electrical signals during the interaction between the analyte and bioreceptor. These aptasensors have low detection limits and high sensitivity with a convenient and rapid detection method [85]. Based on their detection mechanism, electrochemical and piezoelectric approaches are used in electrical aptasensors [13].

In electrochemical aptasensors, an electrical signal change is produced when the aptamer that is immobilized onto an electrode detects its target. In addition, the production of electrical signals can be aided by enzyme catalysis for enzyme-linked aptasensors, or by a field-effect transistor (FET) to detect changes in the electrical charge distribution [13]. One of the electrochemical techniques is voltammetry, which records both the current and potential measurement [63]. Abbaspour et al. developed a dual-aptamer-based sandwich immunosensor to detect *S. aureus* by an electrochemical method through differential pulse stripping voltammetric measurement [86]. In their work, a biotinylated primary aptamer was immobilized on streptavidin-coated magnetic beads to serve as a capture probe, while a secondary aptamer was conjugated with silver nanoparticles (Apt-AgNPs) as a signal probe. The method gave an ultralow limit of detection of 1.0 cfu/mL and was tested in tap- and river-water samples. Besides that, Diba et al. developed a sandwich assay platform consisting of a surface-formed aptamer–protein–antibody complex using an AuNP-modified electrode for the detection of AIV H5N1 viral proteins [87]. The sensing platform managed to get the lowest detectable concentration of 100 fM (100 fM–10 pM) by differential pulse voltammetry. The sensor was further applied for the detection of the virus in human serum samples.

Another technique is known as potentiometry, which measures the potential difference between two electrodes. This technique is commonly used due to its simple operation, portability and low cost [63]. Potentiometric biosensors that are based on carbon nanotubes and aptamers were developed by Zelada-Guillen et al. for label-free detection of *S. aureus* [88]. In this study, aptamers were used as the recognition element of *S. aureus* and a network of single-walled carbon nanotubes (SWCNTs) as the ion-to-electron potentiometric transducer. The aptasensors were constructed by two different approaches—covalent and noncovalent linking—between the aptamers and SWCNTs, providing a difference in the detection limit of 8 × 10^2^ cfu/mL (8 × 10^2^–10^8^ cfu/mL) and 10^7^ cfu/mL (10^7^–10^8^ cfu/mL), respectively. The biosensors were further tested in pig skin samples as a substitute for human skin. In earlier research by the same group, the above approach using SWCNT/aptamer was applied for the potentiometric detection of *E. coli* [89]. A linear detection range up to 10^4^ cfu/mL (4–2.4 × 10^4^ cfu/mL; 4 cfu/mL) was achieved by this aptasensor, and real sample analysis was performed using milk and apple juice samples. Besides that, the impedance technique is another method that measures the impedance change based on the interaction between the targets and bioreceptors that are immobilized on the surface of electrodes, or based on the metabolites produced by bacterial cells from bacterial growth [90]. Bagheryan’s group developed a diazonium-based impedimetric aptasensor for the detection of *S. typhimurium* in apple juice samples [91]. The electrochemical immobilization of the diazonium layer onto screen-printed carbon electrodes (SPEs), followed by chemical immobilization of aminated aptamer, produced a denser aptamer layer with higher sensitivity. The study showed that the developed aptasensor could detect bacteria over a range of 1 × 10^1^–1 × 10^8^ cfu/mL in a buffer medium. More recent research was done to fabricate an electrochemical aptasensor by employing boron-carbon nanorods decorated by nickel nanoparticles (BC-Ni) nanostructured platform [92]. The aptasensor was developed to detect *E. coli* O157:H7 that was present in tap water, juice and fecal samples. With the aid of these highly reactive BC-Ni nanorods, the aptasensor managed to detect the bacteria in a buffer medium with a range of 10^0^–10^5^ cfu/mL, with a detection limit of 10 cfu/mL.

Another type of detection mechanism for electrical aptasensors is piezoelectric transducers. Electrical signals are generated by certain materials due to response to applied mechanical stress. An example of a piezoelectric transducer is a quartz crystal microbalance (QCM). The detectable frequency changes are induced by the aptamers that are fixed on the quartz crystal electrode upon detection of their targets [13]. QCM has been applied in aptasensors to detect the Tat protein of the HIV-1 virus [93], and AIV H5N1 [94]. Minunni et al. developed an RNA-based aptasensor for the Tat protein of HIV-1. The aptamer was immobilized on the gold electrode of the quartz crystals, and the sensor managed to get a detection limit of 0.25 mg/L. On the other hand, Wang and Li developed a hydrogel-based QCM aptasensor to detect AIV H5N1 with a detection limit of 0.0128 HAU. The aptamer was hybridized with an ssDNA, which was then cross-linked with a polymer hydrogel and fixed on the gold surface.

Overall, the research described above demonstrates the successful application of aptamers in biosensor platforms by either optical or electrical principles. Real sample analyses are conducted to demonstrate the feasibility of the developed aptasensors for microbial detection. Table 2 briefly summarizes the detection methods, target microorganisms, detection range and limits in buffer medium, and analyzes real samples of aptamer-based biosensors.

## 5. Applications and Limitations of Aptamers

The major applications of aptamers are in the development of target cell-specific molecular probes and drug-delivery systems [1]. DNA aptamers can work along with DNA origami in delivering drugs to target cells [95]. DNA origami is a three-dimensional box encapsulating drug molecules, while aptamers serve as the lock parts on the box. When the aptamers bind to the target cells, the origami box will be unlocked to expose the drug molecules to their targets, thus completing the drug-delivery system.

The other application of aptamers is that they can be developed as diagnostic tools [10]. Aptamers can be easily combined with techniques such as flow cytometry or nanoparticle-based sensing for better diagnostic purposes [96]. Aptamers play a huge role in the diagnosis of cancer and cardiovascular diseases. In addition, aptamers are applied as small-molecule therapeutic agents in two ways. First, they act as antagonists by inhibiting protein–protein interactions. Second, they act as agonists by increasing the ability of target proteins. According to Zhuo et al., only one aptamer known as Macugen was approved for clinical use to treat neovascular wet age-related macular degeneration (AMD) [10].

However, despite the advantages, aptamers have certain drawbacks that limit their in vivo therapeutic potential. One of the drawbacks is nuclease degradation, which causes the in vivo half-lives of aptamers to be less than 10 min. Modifications have been done at nucleic acid sites, such as the ends of the nucleic acid chain, sugar rings of nucleoside, and phosphodiester linkage, to resist nuclease degradation [10]. For example, the 3′-end of the aptamer is capped with inverted thymidine or biotin to avoid degradation of 3′-exonuclease.

Another drawback is that aptamers can be easily excreted by renal filtration, due to their short diameter (<5 nm) and small mass (6–30 kDa). Hence, a large compound, such as cholesterol [97] or protein [98], is attached to the 5′-end of aptamers to enlarge the aptamer size. For example, a 29 nucleotide-long 2′-F pyrimidine-modified RNA aptamer was attached with cholesterol to form a cholesterol-conjugated aptamer (chol-aptamer). Lee et al. found that the modified aptamer showed high resistance to nuclease degradation with a longer half-life [97].

On the other hand, recent advances have been done to improve aptamer-selection technology. A review article by Blind and Blank showed that next-generation sequencing (NGS) is used for the analysis of random libraries at the beginning of the SELEX process [99]. The purpose is to optimize the SELEX libraries for aptamer selection as the libraries may consist of millions of sequences. An optimized SELEX library with a balanced nucleotide distribution containing all four bases increases the chance to select aptamers with desired binding activities. The data obtained from NGS give a view of the distribution of nucleotide bases in the random library, thus allowing the adjustment of protocol to obtain the desired library.

Furthermore, several approaches have been made for the improvement of the stability of aptamers. First of all, modifications are made to nucleic acid-based aptamers (DNA, RNA) to overcome nuclease-mediated cleavage [100]. Some examples of modifications on DNA aptamers are done on linkage (addition of an extra guanine base to the 3′-end) [101], and also on the sugar ring and bases (addition of a methylene linkage between the 2′-oxygen and the 4′-carbon of the sugar ring) [102]. For RNA aptamers, the most common modification is on the 2′-position of the sugar ring, for example, the addition of a 2′-OH group [103], and substitution with 2′-F on pyrimidine [104]. Wang et al. mentioned that several modifications such as the 3′-inverted T capping, phosphorothioate linkage and 2′-fluoro substitution, are effective on both DNA and RNA aptamers as they have similar structures [100].

## 6. Conclusions

In summary, the development of aptamers through SELEX technology has the potential to become an alternative to the traditional approaches for diagnostic and therapeutic purposes. As described in this review, aptamers and SELEX technology have promising advantages. The aptamers are widely applied to detect pathogenic microorganisms including bacteria, viruses and unicellular parasites, as well as mammalian cells. In addition, they are utilized in biosensor platforms through optical or electrical principles for more advanced detection methods. The general SELEX scheme and cell-internalizing SELEX technology have been discussed to give an overview of the SELEX protocol.

Despite certain limitations of aptamers, improvement strategies have been done to enhance the aptamer-selection technology. At present, although this technology has significant contributions in diagnostic and therapeutic fields, extensive research would be required to develop more aptamers for practical use. For instance, aptamers could be applied in commercial kits to provide simple, rapid, and low-cost detection. The application of aptamers should not be limited to biomedical fields, but also used in agriculture and aquaculture for detection of food-borne pathogens. In addition, the selection of aptamers could be improved to detect multiple targets at the same time instead of a certain target. However, the sensitivity and stability of aptamers remain as challenges that need to be overcome for better analytical performance in the future. Overall, aptamers have their robustness and unique qualities that can be further explored and applied for the benefit of mankind.

## Figures and Tables

**Table 1:** Pathogenic bacteria, viruses and unicellular parasites targeted by aptamers.

Target Microorganisms	Nucleic Acid Pool	Dissociation Constant (Kd)	References
Bacteria			
*Escherichia coli* O157:H7	DNA	10.30 nM	[6]
	DNA	107.6 ± 67.8 pM	[7]
	DNA	9.04 ± 2.80 nM	[34]
*Mycobacterium tuberculosis* H37Rv	DNA	-	[20,21]
*Mycobacterium tuberculosis*	DNA	Nanomolar (nM) range	[35]
*Salmonella typhimurium*	DNA	6.33 ± 0.58 nM	[17]
	DNA	0.360 ± 0.103 nM	[36]
*Salmonella typhimurium*, *Salmonella enteritidis*	DNA	Nanomolar (nM) to micromolar (µM) range	[18]
	DNA	25 nM, 7 nM	[37]
*Staphylococcus aureus*	DNA	35 nM, 129 nM	[38]
*Vibrio alginolyticus*	DNA	14.31 ± 4.26 nM, 90.00 ± 13.51 nM	[39]
	DNA	27.5 ± 9.2 nM	[40]
Viruses			
Avian influenza H9N2 virus	DNA	Nanomolar (nM) range	[22]
Avian influenza H5N1 virus	RNA	-	[23]
Avian influenza H1N1 virus	DNA	55.14 ± 22.40 nM	[41]
Hirame rhabdovirus (HIRRV)	RNA	-	[29]
Human immunodeficiency virus (HIV)	RNA	-	[24]
	RNA	0.15 nM	[25]
	DNA	-	[26]
Red-spotted grouper nervous necrosis virus (RGNNV)	DNA	Nanomolar (nM) range	[31]
Severe acute respiratory syndrome coronavirus 2 (SARS-CoV-2)	2′-fPy-RNA	Picomolar (pM) range	[27]
	DNA	0.118 ± 0.033–85.610 ± 14.219 nM	[28]
Singapore grouper iridovirus (SGIV)	DNA	-	[30]
Unicellular parasites			
*Leishmania infantum*	DNA	0.94 ± 0.19 nM	[42]
*Plasmodium falciparum*	2′-fPy-RNA	-	[33]
	DNA	42 nM	[43]
*Trypanosoma brucei*	2′-NH_2_-RNA	70 ± 15 nM	[44]
*Trypanosoma cruzi*	2′-fPy-RNA	40–400 nM	[32]

**Table 2:** Various detection methods by aptamer-based biosensors for microbial detection.

Detection Method	Target Microorganisms	Detection Range	Limit of Detection	Real Samples	References
Optical					
Fluorescence	*Escherichia coli*	~100/s detection rate on single *E. coli* cells	-	-	[72]
Fluorescence	*Pseudomonas aeruginosa*	1.28 × 10^3^–2.00 × 10^7^ cfu/mL	100 cfu/mL	Drinking water, orange juice, popsicle	[73]
Chemiluminescence	*Escherichia coli* O157:H7	10^4^–10^7^ cfu/mL	4.5 × 10^3^ cfu/mL	Skim milk	[75]
Colorimetric	*Salmonella typhimurium*	25–10^5^ cfu/mL	10 cfu/mL	Milk	[77]
Colorimetric	*Shigella flexneri*	10^2^–10^6^ cfu/mL	80 cfu/mL	Salmon	[79]
Colorimetric	*Staphylococcus aureus*	50–10^4^ cfu/mL	20 cfu/mL	Milk	[78]
Surface plasmon resonance (SPR)	AIV H5N1	0.128–1.280 HAU	0.128 HAU	Poultry	[81]
Surface-enhanced Raman scattering (SERS)	*Salmonella typhimurium*	10^1^–10^5^ cfu/mL	4 cfu/mL	Pork	[83]
Surface-enhanced Raman scattering (SERS)	*Salmonella typhimurium, Staphylococcus aureus*	10^2^–10^7^ cfu/mL	15 cfu/mL; 35 cfu/mL	Pork	[84]
Electrical					
Differential pulse voltammetry	*Staphylococcus aureus*	10–1 × 10^6^ cfu/mL	1 cfu/mL	Tap and river water	[86]
Differential pulse voltammetry	AIV H5N1	100 fM–10 pM	100 fM	Human serum	[87]
Potentiometry	*Escherichia coli*	4.0–2.4 × 10^4^ cfu/mL	4.0 cfu/mL	Milk, apple juice	[89]
Potentiometry	*Staphylococcus aureus*	8 × 10^2^–10^8^ cfu/mL;10^7^–10^8^ cfu/mL	8 × 10^2^ cfu/mL; 10^7^ cfu/mL	Pig skin	[88]
Impedance	*Escherichia coli O157:H7*	10^0^–10^5^ cfu/mL	10 cfu/mL	Tap water, juice, fecal	[92]
Impedance	*Salmonella typhimurium*	1 × 10^1^–1 × 10^8^ cfu/mL	6 cfu/mL	Apple juice	[91]
Piezoelectricity	AIV H5N1	-	0.0128 HAU	-	[94]
Piezoelectricity	Tat protein of HIV-1 virus	0–1.25 mg/L	0.25 mg/L	-	[93]

## Data Availability

Not applicable.
